# Research on the Mechanical Properties and Durability Against Freeze–Thaw of Latex-Modified OPC-Based Materials

**DOI:** 10.3390/molecules31193535

**Published:** 2026-10-04

**Authors:** Lang Jin, Zixiao Hong, Feixiang Chen, Guozhi Zhang, Xiang Su, Luyan Wang

**Affiliations:** 1 CCCC Second Harbor Engineering Company Ltd., Wuhan 430040, China; 2 CCCC Highway Bridge National Engineering Research Centre Co. Ltd., Beijing 100088, China; 3 School of Chemistry and Chemical Engineering, University of Jinan, Jinan 250022, China

**Keywords:** natural rubber latex, mechanical properties, freeze–thaw resistance, OPC

## Abstract

The intrinsic brittleness and poor crack resistance of OPC-based materials severely limit their service durability under complex environments. In this study, a composite latex polymer was fabricated via an ambient-temperature APS/STS redox initiation system, using N,N′-methylenebisacrylamide (MBA) as a cross-linking agent and incorporating reactive SiO_2_ and MgO as synergistic reinforcing fillers. The composite latex was incorporated into OPC paste, and its effects on the mechanical strength, freeze–thaw resistance, water permeability, and microstructural characteristics of the hardened composites were systematically evaluated. The results show that, at an optimal dosage of 0.2 wt%, the 28-day flexural and compressive strengths of the modified OPC specimens were respectively increased by 16% and 18% compared with the plain cement control, while the strength loss after freeze–thaw cycling was effectively controlled within 10% and the maximum impermeability pressure rose from 2.9 to 3.2 MPa. FTIR and XRD analyses confirmed that the incorporation of the latex polymer did not alter the phase composition of the OPC hydration products but promoted the generation of C-S-H gel. SEM observations further revealed that the polymer filled internal micro-pores and microcracks, yielding a denser organic–inorganic composite microstructure. The improved performance may be attributed to the potential synergistic interplay among APS/STS-triggered cross-linking, pozzolanic reaction of reactive SiO_2_, and the micro-expansion effect of MgO. This work provides a feasible technical approach for the development of high-toughness and frost-resistant latex-modified OPC-based materials.

## 1. Introduction

Cement production is a major contributor to global anthropogenic CO_2_ emissions, accounting for approximately 8% of the global total. With the escalating urgency of climate change and the growing demand for infrastructure, the construction industry faces immense pressure to reduce its carbon footprint. Consequently, enhancing the durability and service life of cement-based materials in harsh environments has emerged as a critical strategy for sustainable development, as it significantly reduces the need for frequent repairs and reconstruction. Cement-based materials are inorganic non-metallic composites fabricated by hydrating and hardening hydraulic cementitious materials (e.g., Portland cement) mixed with water, aggregates, and functional admixtures [1]. Recent studies have further illustrated that the calcination process of natural colemanite powder can significantly influence the compressive strength of concrete while offering a route to reduce clinker content [2], and that processed tea waste ash can serve as a sustainable SCM that promotes hydration products and improves the mechanical performance of hybrid cement systems [3]. Covering ordinary mortar, concrete, and high-performance modified cementitious composites, they have been widely applied in civil engineering infrastructure. Nevertheless, the intrinsic high porosity and brittle cracking characteristics of cement-based materials severely limit their long-term service durability under harsh environmental conditions [4]. Cement-based materials are inherently low in tensile strength and fracture toughness, rendering them prone to crack initiation and propagation under tensile, flexural, and complex loading conditions. In practical engineering service, temperature variation, drying shrinkage, and external mechanical loads further accelerate crack development [5]. The formed cracks not only degrade the structural bearing capacity but also provide permeable channels for harmful substances such as water and deicing salts to penetrate into the cement matrix [6,7].

Among various environmental stressors, freeze–thaw action is recognized as one of the most severe deterioration factors for cement-based materials in cold regions [8,9]. During repetitive freezing and thawing cycles, the volumetric expansion (approximately 9%) of water as it transforms into ice within capillary pores generates significant internal tensile stresses. When these stresses exceed the local tensile strength of the cement matrix, micro-cracks initiate and progressively propagate, leading to spalling, scaling, and ultimately structural failure [10,11]. The situation is further aggravated by the simultaneous application of deicing salts in pavement engineering, which exacerbates surface scaling through osmotic pressure and thermal shock effects [12]. Therefore, improving the freeze–thaw resistance of cement-based materials is of paramount importance for extending the service life of infrastructure in cold regions, reducing maintenance costs, and minimizing the environmental burden associated with premature reconstruction. It is also worth noting that cement manufacturers are progressively replacing Portland cement with Supplementary Cementing Materials (SCMs)—such as natural pozzolans, fly ash, calcined clays and shales, and silica fume (the most reactive pozzolan among conventional SCMs)—to mitigate the carbon footprint of cement plants. Among these materials, nano-SiO_2_ exhibits higher pozzolanic activity than silica fume. It is also noteworthy that low dosages of polymer latex (around 0.2 wt% of the binder) have been reported in the literature to be effective in refining the pore network and improving the impermeability of cementitious systems without severe strength loss [13,14,15]. In this context, the reactive nano-SiO_2_ and silica fume used in this study are siliceous pozzolans in nature, and silicic in chemical character (Talero [16]). Under identical dosage and experimental conditions, reactive nano-SiO_2_ exhibits higher pozzolanic activity than silica fume. Although the reactive nano-SiO_2_ is introduced as a reinforcing filler rather than a clinker replacement, its pozzolanic activity is still expected to satisfy the requirements of EN 197-1 for silica-fume-bearing OPC blends at later ages [16,17,18].

**Figure 1:**
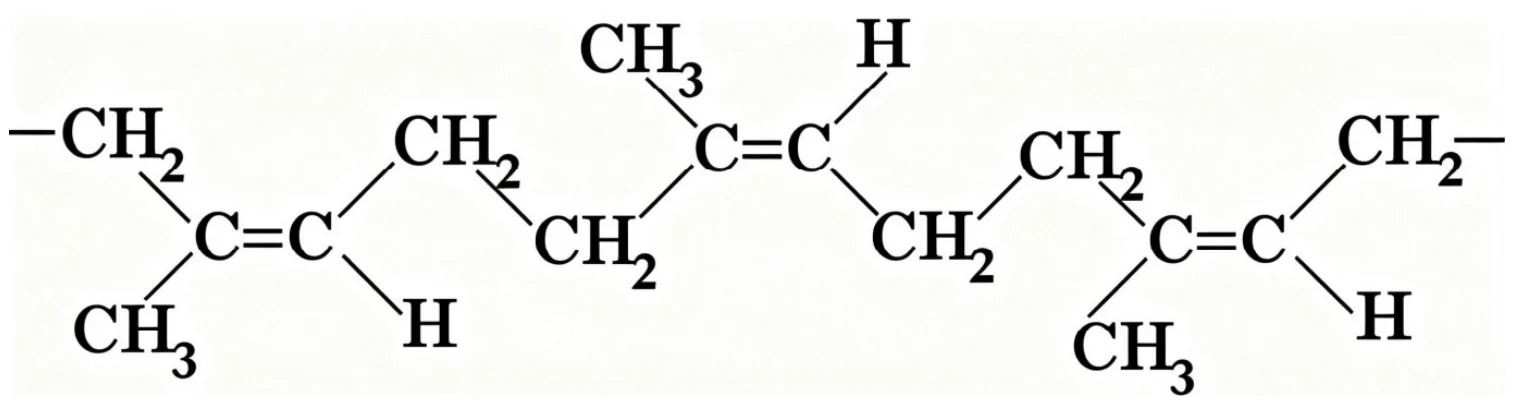
Schematic molecular structure of natural rubber (cis-1,4-polyisoprene) from natural rubber latex (NRL).

Natural rubber latex (NRL), with cis-1,4-polyisoprene as its core component, occupies an irreplaceable position in the field of high-performance cement-based modified materials due to its unique oil-in-water (O/W) colloidal dispersion system. It endows the materials with exceptionally high elasticity and resilience, as illustrated in Figure 1. Latex polymer modification has been proven to be an efficient and promising reinforcement strategy for cement-based materials. The flexible polymer film formed inside the cement matrix can effectively improve fracture toughness, impermeability, and freeze–thaw resistance, exhibiting broad application prospects in pavement maintenance, hydraulic structure protection, and high-performance waterproofing engineering [19,20]. Previous studies have verified the effectiveness of NRL modification. Buritatum et al. [21] reported that NRL incorporation effectively improves the compressive and flexural strength of cement-stabilized pavement base materials and alleviates pavement deterioration under dry-wet alternating conditions in tropical regions. Al-Akhras and Smadi [22] demonstrated that rubber particle densification effect optimizes the pore structure of mortar matrix, thereby improving the freeze–thaw durability of modified cement composites. Savas et al. [23] replaced cement with rubber particles at different mass ratios and found that 10% and 15% rubber dosage significantly enhanced the freeze–thaw resistance of concrete, with the durability index improved by approximately 60% after 300 freeze–thaw cycles. However, conventional NRL-modified cementitious systems still suffer from prominent limitations, including weak interfacial bonding strength between rubber polymer and cement matrix, high hydrophilicity of polymer films, and delayed cement hydration that causes adverse late-age strength attenuation [24]. Traditional physical blending methods fail to eliminate molecular-level interfacial incompatibility between organic polymers and inorganic cement matrices, resulting in limited performance improvement of modified composites [25]. In recent years, chemical functionalization and cross-linking modification have become mainstream research directions to enhance latex activity and optimize polymer-cement interfacial microstructure, which provides a feasible way to break through the performance bottleneck of traditional latex modification [13]. In agreement with these reports, the optimum latex dosage identified in the present work (0.2 wt% relative to OPC) lies within the low-dosage window that previous studies have found beneficial for pore densification and durability enhancement of latex-modified OPC systems [14,15].

In view of the above limitations of conventional modification techniques, this study proposes a novel organic–inorganic synergistic modification strategy for natural rubber latex incorporating nano-SiO_2_, and systematically evaluates the mechanical properties and freeze–thaw resistance of the resulting latex-modified cement-based composites. This work specifically focuses on performance under cold-region conditions, and can provide a theoretical basis and technical reference for the performance optimization and engineering application of high-toughness and frost-resistant latex-modified OPC-based materials [26].

## 2. Results and Discussion

### 2.1. Effects of SiO_2_ and MgO on Latex Polymer Film Properties

A gravity tensile test was performed to characterize the tensile deformation capacity of polymer films. Specifically, a 500 g weight was suspended at the lower end of the prepared polymer film to induce free stretching under gravity, and the corresponding elongation was recorded. The maximum stretch ratio was defined as the ratio of the ultimate stretched length to the initial original length of the polymer film.

**Figure 2:**
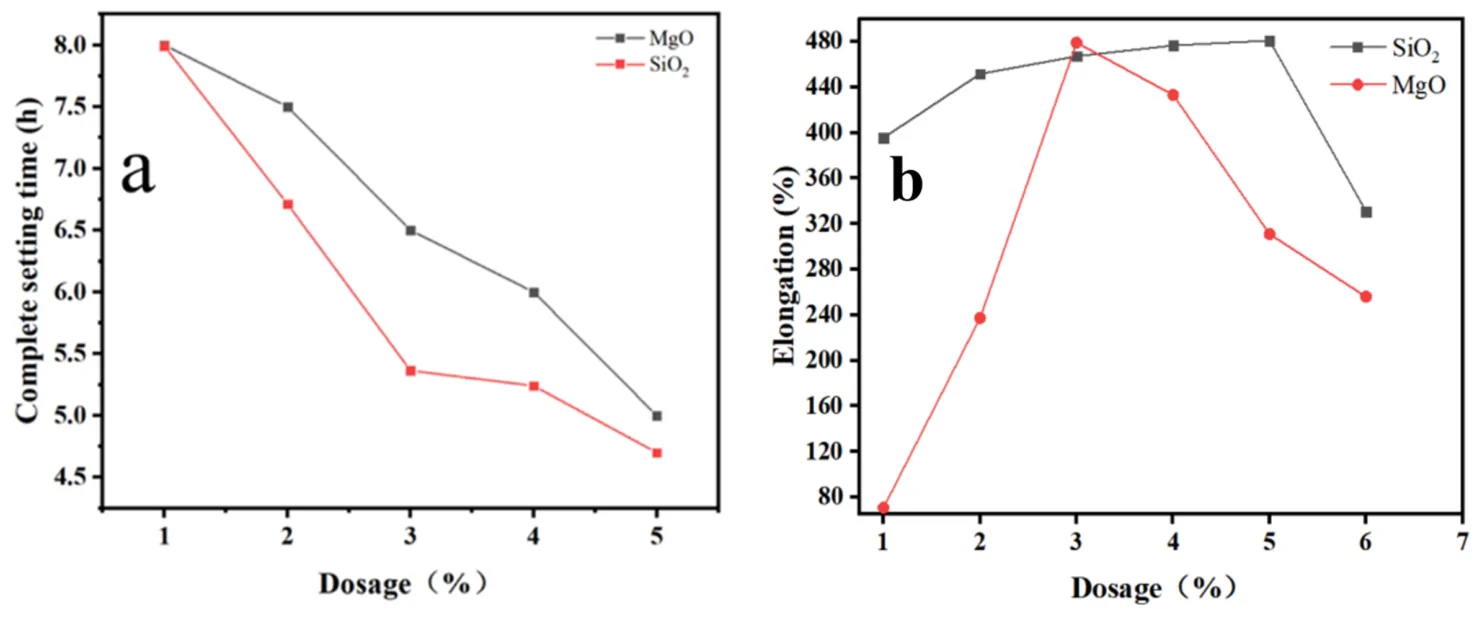
Effects of the incorporation of SiO_2_ and MgO on the properties of the polymer film: (**a**) curing time; (**b**) tensile properties.

Figure 2a presents the variation in film curing time with the incorporation of SiO_2_ and MgO, where the dosages of natural rubber latex (NRL), APS, STS, and MBA were kept constant. With the increasing addition of nano-SiO_2_ and MgO, the film-forming time of modified NRL polymer gradually decreases. This accelerated film-forming behavior is closely related to the changed colloidal stability of the NRL system. The original latex system maintains stable dispersion relying on the electric double layer structure formed by anionic emulsifiers adsorbed on rubber particle surfaces. Nano-SiO_2_ with abundant surface silanol groups plays a dual regulatory role [27]. On the one hand, nano-SiO_2_ can adsorb fatty acid-based emulsifiers in the latex, destroying the protective film on rubber particles. On the other hand, the dissociation of surface silanol groups releases trace hydrogen ions, which slightly reduces the local pH value, neutralizes the negative charge on the latex particle surface, and thereby promotes latex flocculation [28]. Meanwhile, MgO gradually hydrates in the aqueous environment to generate magnesium hydroxide. The increased ionic concentration further raises the electrolyte level of the system, accelerates latex demulsification and particle agglomeration, and effectively shortens the coagulation and film-forming period of the modified latex [29].

Figure 2b shows the tensile performance evolution of polymer films with varying dosages of SiO_2_ and MgO. All tensile tests were performed using at least three parallel specimens, and the reported values represent arithmetic averages. Overall, the tensile properties of modified films present a typical trend of increasing first and then decreasing with the continuous addition of inorganic fillers. In the single MgO modification group without SiO_2_ incorporation, the elongation at break increases first and then declines with the rise in MgO dosage. The maximum elongation at break of 479.2% is achieved at a MgO dosage of 3%. On this basis, with the MgO content fixed at 3%, further incorporation of SiO_2_ gradually improves the film ductility, and the peak elongation at break reaches 481% at a SiO_2_ dosage of 5%. Considering the limited performance improvement among some groups, this mixing ratio is tentatively taken as a reference only based on the present single-factor results. It should be noted that the dosages of SiO_2_ (5 wt%), MgO (3 wt% of the latex emulsion mass), and MBA (0.031 g per 10 g of latex emulsion) were determined on the basis of single-factor screening experiments; the underlying justification for the associated setting time variation, however, requires further verification through orthogonal design and comprehensive mechanistic characterization.

### 2.2. Analysis of Polymerization and Film Formation Mechanism

**Figure 3:**
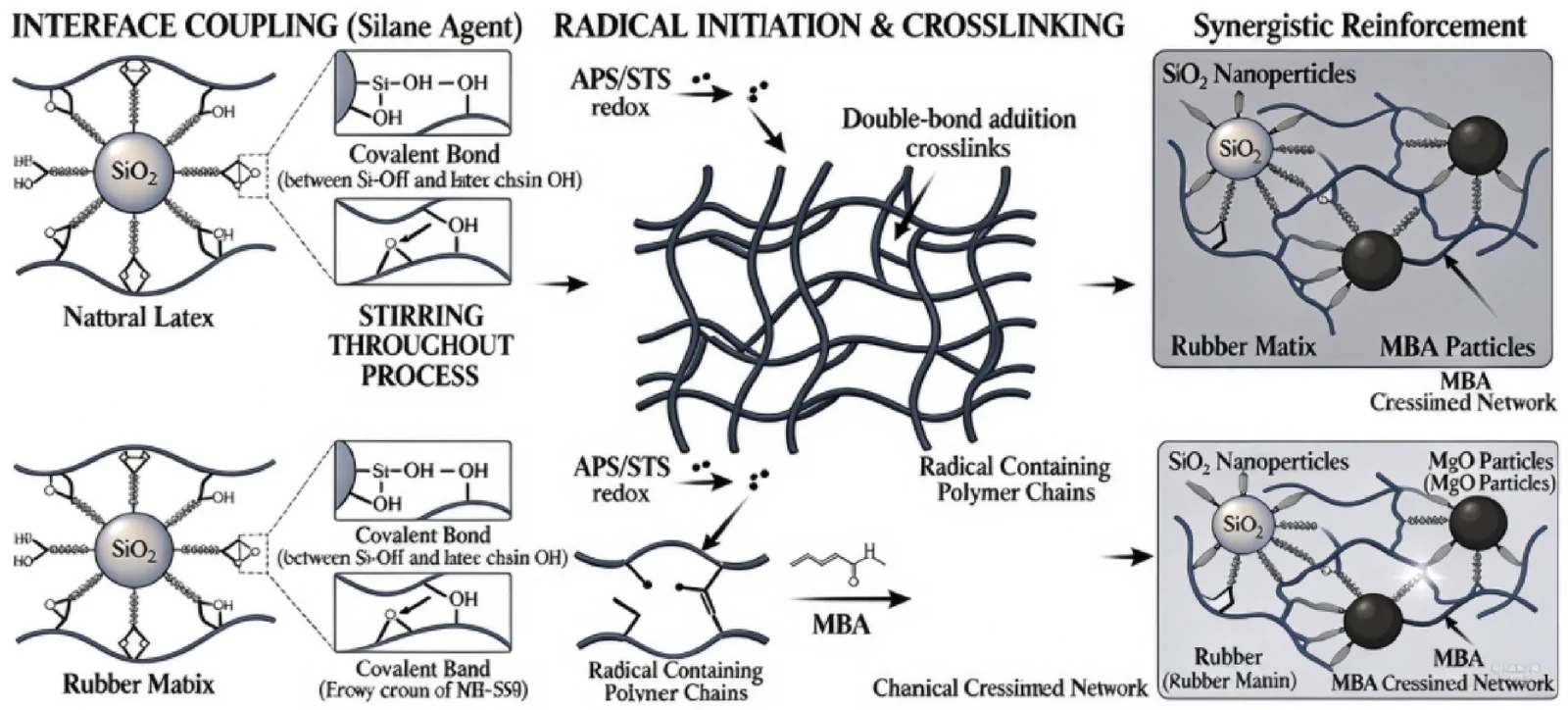
Schematic diagram of the film formation mechanism for the latex polymer.

The polymerization mechanism of the polymer is shown in Figure 3.Both ammonium persulfate (APS) and sodium thiosulfate (STS) are water-soluble initiators that can undergo a single-electron transfer reaction in the aqueous phase of natural rubber latex (NRL). This reaction generates two highly active free radicals, including sulfate radicals (SO_4_^•−^) and thiosulfate radicals (S_2_O_3_^•−^). The produced free radicals can effectively attack the carbon-carbon double bonds (C=C) on natural rubber molecular chains and activate the terminal vinyl groups of the N,N′-methylenebisacrylamide (MBA) cross-linker [30,31,32,33]. Accordingly, graft copolymerization and intermolecular cross-linking reactions are simultaneously triggered within the latex system [34]. As MBA molecules contain dual reactive vinyl groups, they can act as chemical bridging units to connect adjacent polyisoprene molecular chains, forming a stable cross-linked network structure in the modified latex film [35]. This cross-linked structure plays a decisive role in optimizing the mechanical performance and flexible deformation capacity of polymer films. The role of this cross-linked network structure is primarily manifested in restricting the mobility of polymer molecular chains, thereby reducing the swelling ratio and solubility of the film in water or chemical solutions. This is critical for the long-term durability of cement-based materials in humid or corrosive environments.

The APS/STS redox initiation system exhibits a prominent polymerization acceleration effect. Without the redox initiator, the complete curing time of pure latex emulsion lasts 6–8 h. With the incorporation of the APS/STS system (with the dosages of SiO_2_, MgO and MBA kept constant), the curing period is shortened to 3–4 h. The dramatically reduced curing duration verifies that free radical initiation can efficiently activate chain polymerization reactions and achieve high monomer conversion in a short time [36].

The dosage of MBA cross-linker dominates the cross-linking density and macroscopic mechanical performance of modified films, presenting a typical dual regulatory effect [37]. In this study, the optimal MBA dosage is determined as 0.031 g per 10 g of latex emulsion, which endows the polymer film with outstanding toughness. Excessively increasing the MBA dosage to 0.062 g and 0.093 g significantly deteriorates film toughness and even causes surface cracking. Excessive cross-linking forms dense network structures that severely constrain the movement and sliding of rubber molecular chain segments [38]. Under external tensile stress, the restricted molecular chains fail to complete effective orientation and rearrangement, resulting in a continuous reduction in film elongation at break [39]. In general, a moderate cross-linker dosage can balance cross-linking density and molecular chain flexibility, which is the fundamental principle for determining the optimal MBA dosage in this modification system.

Overall, the APS/STS redox system accelerates radical polymerization and cross-linking kinetics, while the moderate addition of MBA constructs a uniform and stable cross-linked network. The synergistic effect of initiation activation and cross-linking regulation effectively optimizes the microstructure and comprehensive mechanical properties of NRL polymer films [40].

### 2.3. Mechanical Strength

**Figure 4:**
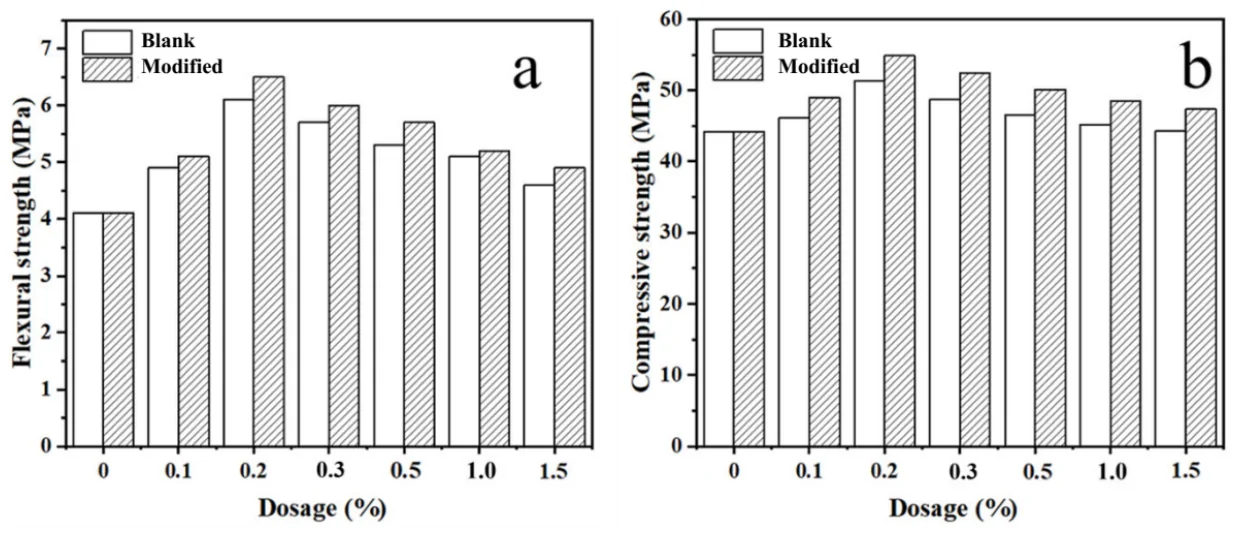
Effect of latex polymer dosage on the 28-day mechanical strength of cement paste: (**a**) flexural strength; (**b**) compressive strength. Blank: neat OPC paste without any admixtures. Modified: OPC paste incorporated with latex polymer.

The optimized latex-modified polymer emulsion, prepared with fixed dosages of APS, STS, MBA, SiO_2_, and MgO, was mixed into fresh cement paste at different mass fractions relative to cement. After demolding, all specimens were cured in a constant-temperature-humidity chamber for 28 days for full hydration, followed by flexural and compressive strength measurements. Figure 4 illustrates the 28-day mechanical performance of OPC composites at different latex emulsion dosages. Compared with neat OPC paste, all modified groups showed enhanced mechanical properties. The strengthening effect first increased with the latex dosage and reached a maximum at 0.2%, where the flexural strength increased by 16% and the compressive strength increased by 18% relative to the neat OPC control. Further increasing the latex dosage beyond 0.2% led to a gradual decline in both flexural and compressive strengths, although the mechanical strengths remained higher than those of the unmodified blank group within the tested dosage range (0.1–1.5%). The 0.2 wt% optimum dosage identified here is consistent with the low-dosage range reported in the literature for latex-modified Portland cement systems, where small polymer additions are sufficient to densify the pore structure and improve impermeability without introducing excessive air voids [14,15]. Compared with commercial polymer modifiers, the self-synthesized composite latex in this work acts as a cost-effective alternative [41].

**Figure 5:**
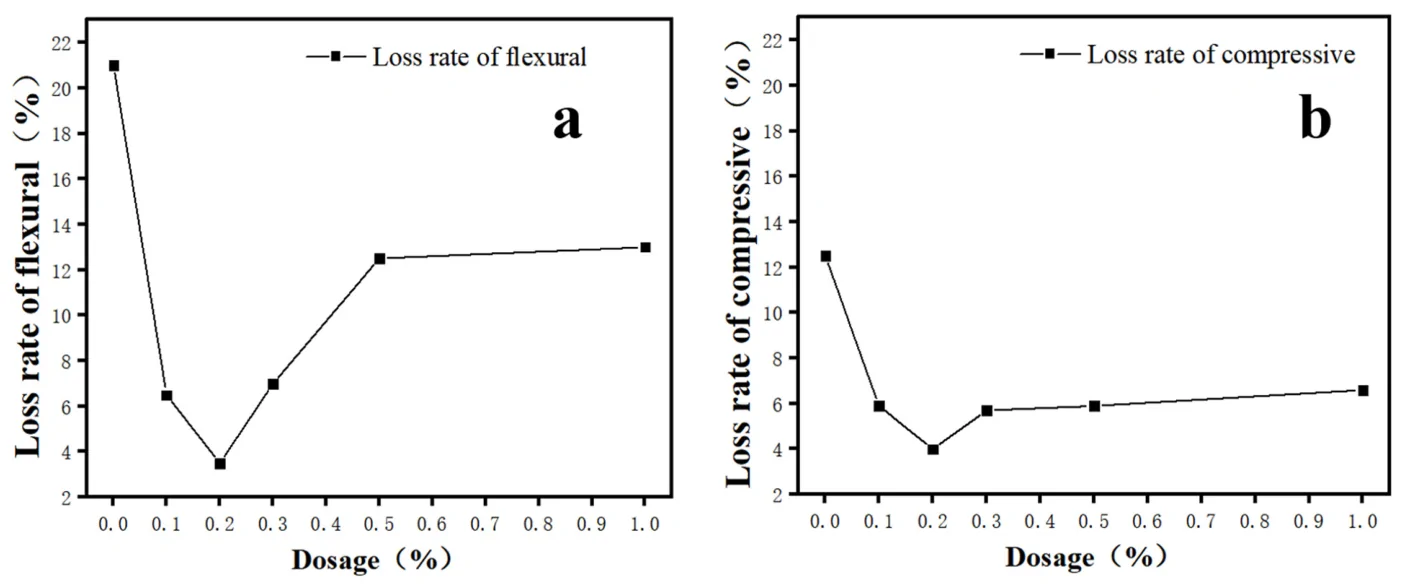
Effect of latex polymer dosage on the mechanical strength of cement after seven freeze–thaw cycles: (**a**) loss rate of flexural strength; (**b**) loss rate of compressive strength.

As can be observed from the mechanical strength loss rates under seven freeze–thaw cycles in Figure 5, the plain OPC paste exhibits substantial flexural and compressive strength loss after freeze–thaw cycles, indicating severe freeze–thaw damage. In contrast, the OPC specimens incorporating the latex polymer show a significantly reduced strength loss rate after freeze–thaw cycles, demonstrating that the addition of the latex polymer emulsion effectively enhances the freeze–thaw resistance of the OPC composites. Notably, the specimens with a 0.2% dosage exhibit the minimum flexural and compressive strength loss after freeze–thaw cycles, possessing the optimal freeze–thaw resistance within the tested cycles. The superior residual strength of the 0.2% group indicates that this dosage provides the best compromise between polymer film formation and OPC hydration, in line with previous observations that optimal latex contents effectively fill capillary pores and enhance interfacial adhesion [14,15].

**Figure 6:**
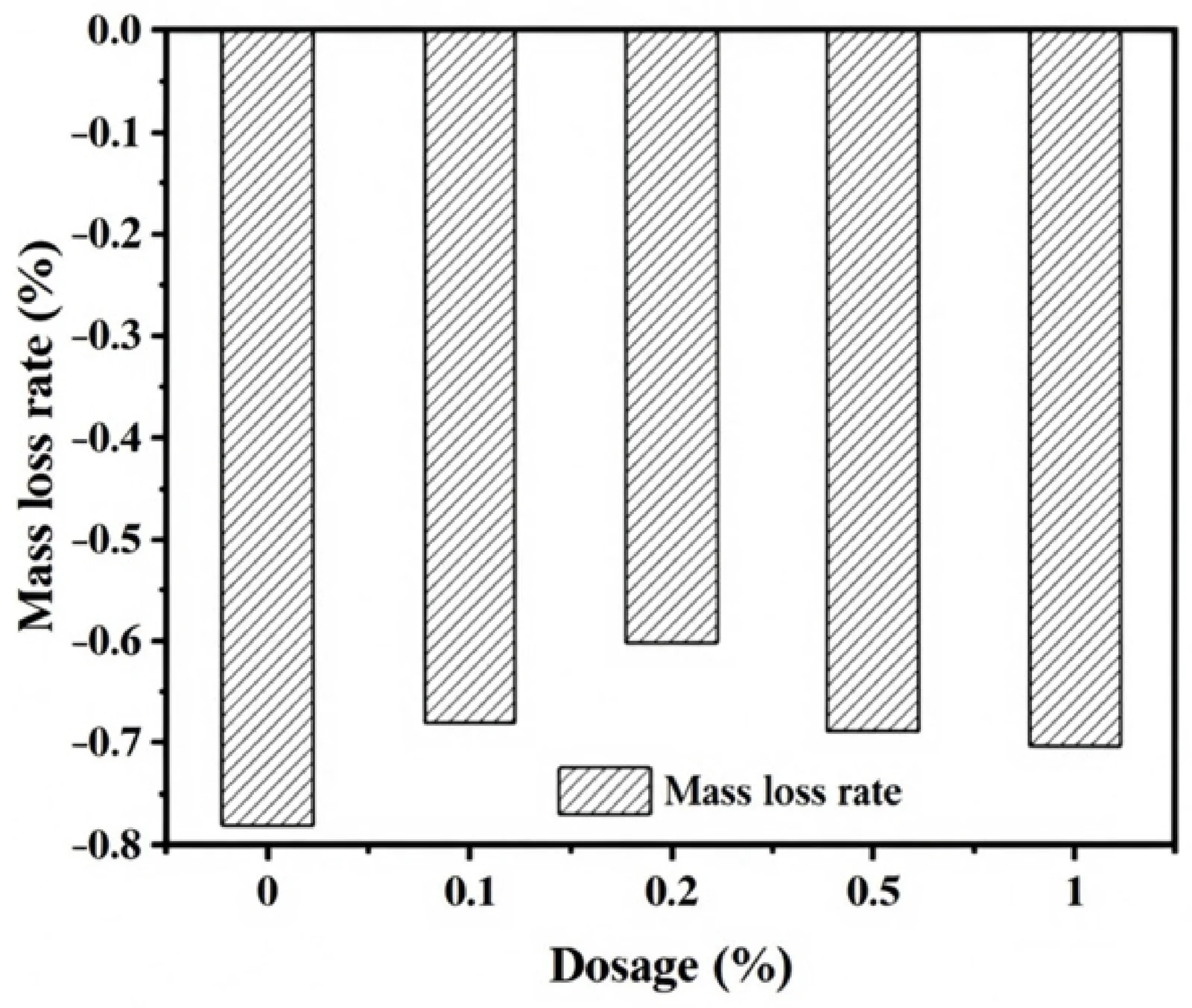
Effect of latex polymer dosage on the mass loss rate of cement specimens subjected to seven freeze–thaw cycles.

As shown in Figure 6, negative mass loss values were observed for all specimens after seven freeze–thaw cycles, which corresponds to a slight mass increment caused by water absorption during cyclic immersion. Although the overall mass variation was insignificant, the 0.2% latex-modified group exhibited the lowest water absorption and the smallest mass change among all tested dosages. This phenomenon indicates that the optimal 0.2% latex dosage can densify the internal pore structure of OPC composites, inhibit water penetration, and consequently endow the material with improved freeze–thaw resistance and structural stability under the employed cycling conditions. Such pore-refinement and adhesion-enhancement effects are consistent with the well-documented observation that substituting appropriate mineral additives into the cement matrix fills the pore structure of cement/concrete and increases interfacial adhesion, thereby improving both mechanical properties and durability. In particular, the substitution of raw ground colemanite has been demonstrated to refine the pore structure and significantly improve the durability of hardened concrete [14].

### 2.4. Setting Time

**Figure 7:**
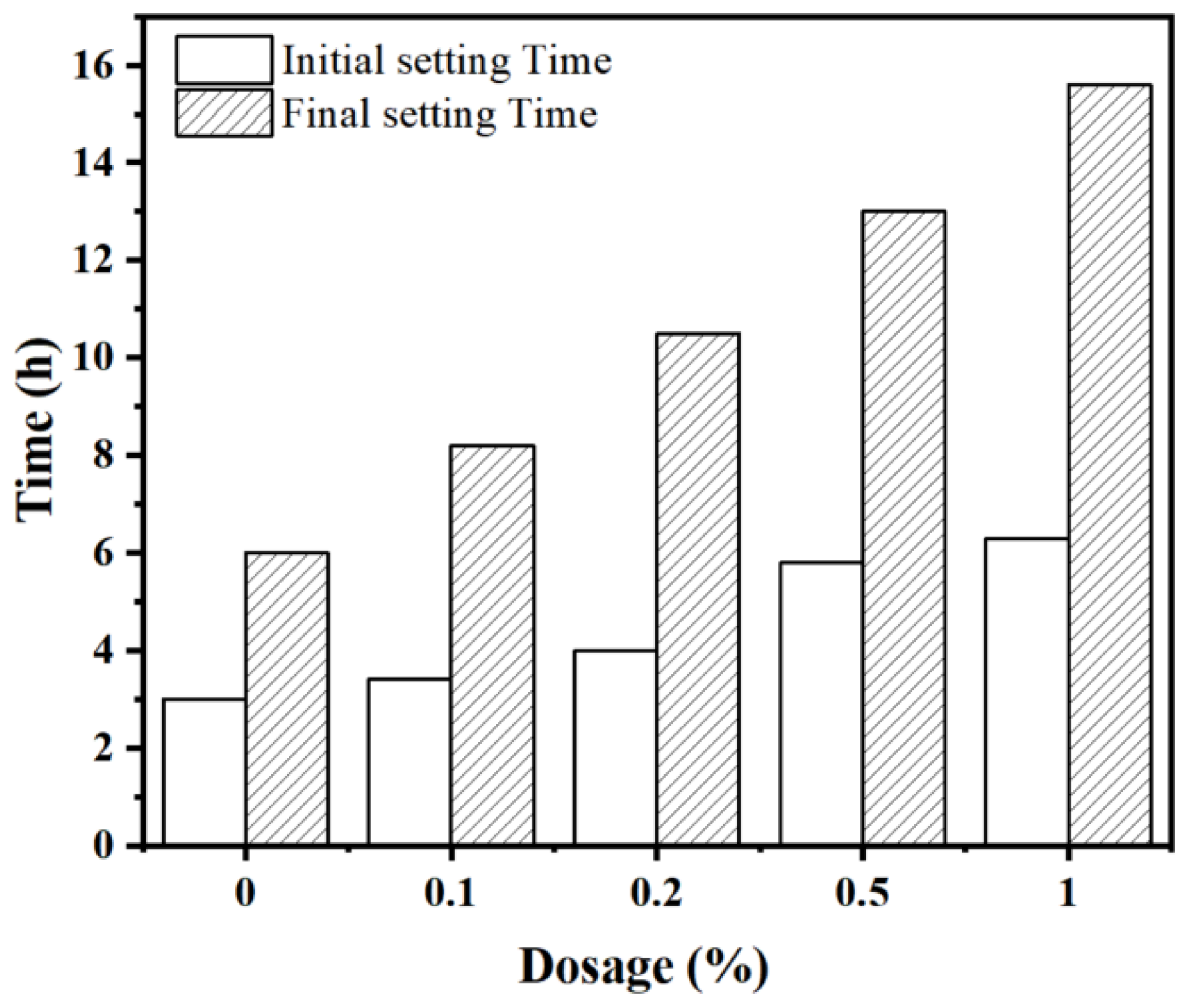
Effect of latex polymer dosage on the setting time of cement paste.

Because polymer additives may alter the hydration kinetics and working performance of OPC paste, the setting time evolution of latex-modified OPC paste was systematically investigated in this section to clarify the influence of the optimized latex polymer on cement early hydration behavior and to evaluate its working adaptability. As presented in Figure 7, compared with plain OPC paste, the incorporation of latex polymer gradually prolongs both the initial and final setting times of OPC paste with increasing dosage. This demonstrates that the modified latex polymer can slow down the early hydration process of cement, and such delaying behavior is positively correlated with polymer dosage [42]. This phenomenon originates from the adsorption of latex polymer particles on the surface of unhydrated OPC grains. With the continuous consumption of free water during cement hydration, the adsorbed polymer particles gradually aggregate and coalesce, forming a continuous or semi-continuous hydrophobic film covering cement particles and hydration product surfaces. The formed polymer film acts as a physical barrier that inhibits the inward penetration of external water and restricts the outward diffusion of hydration ions. At the macroscopic level, this barrier effect delays the early hydration reaction rate, thereby extending the initial and final setting times and slowing the early strength development of OPC paste.

Despite the noticeable delaying effect on early cement hydration, the modified latex polymer exhibits a prominent positive modification effect on the microstructural and long-term macroscopic performance of cement-based materials. During continuous cement hydration, deposited polymer films interweave with hydration products such as C-S-H gel to form a dense organic–inorganic composite structure [43]. This composite structure can effectively fill capillary pores inside the cement matrix, reduce material permeability, and improve chemical corrosion resistance [44]. Meanwhile, the flexible polymer component imparts excellent deformation ability and crack resistance to the rigid cement matrix, optimizing the inherent brittle defects of cement materials and enhancing interfacial bonding performance. Overall, the synergistic optimization of pore structure and mechanical toughness significantly improves the long-term service stability and durability of latex-modified cement composites. These effects collectively contribute to a denser microstructure and lower permeability, as has also been observed for other mineral-additive systems in which pore filling and ITZ refinement improve long-term durability [14,15].

### 2.5. Waterproofing Performance Test of Cement

**Figure 8:**
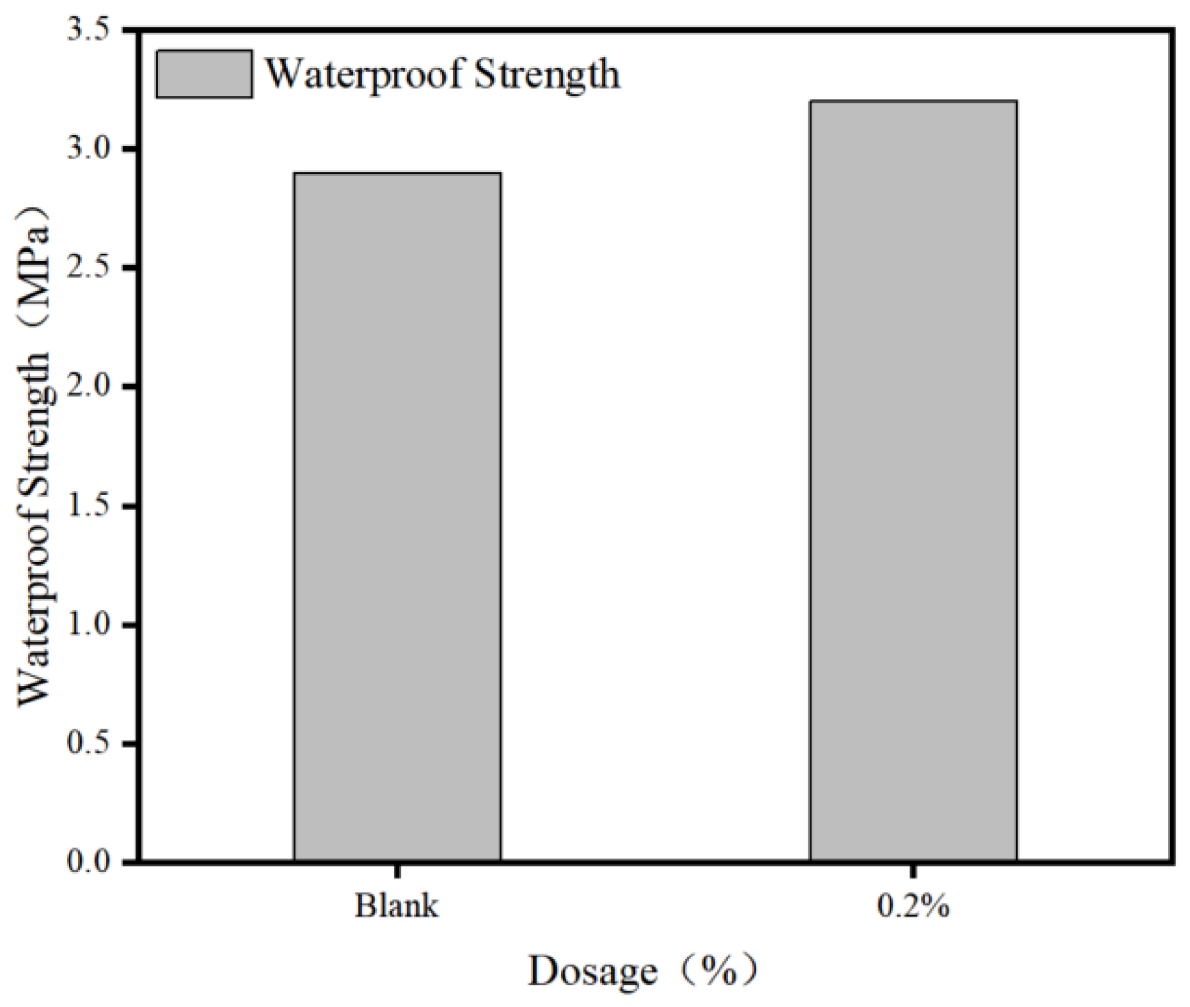
Effect of optimal latex polymer incorporation on the impermeability of cement specimens.

Water permeability is a key indicator determining the durability of cementitious composites, and the impermeability of cement specimens modified with the optimal latex formula was therefore further tested in this section. All specimens were prepared using the latex polymer with optimized SiO_2_ and MgO dosages, and the optimal 0.2% latex dosage was adopted for cement modification. As shown in Figure 8, the maximum impermeability pressure of plain cement paste is 2.9 MPa, while the 0.2% latex-modified cement specimen achieves an impermeability pressure of 3.2 MPa, corresponding to a 10% enhancement in water resistance. The tested results confirm that the appropriate incorporation of modified latex can effectively optimize the internal pore structure of cement matrix, cut off water penetration channels, and significantly improve the impermeability of cement-based materials. The improved impermeability can effectively hinder the invasion of external water and harmful corrosive media, maintain the structural integrity and internal stability of cement composites, and further guarantee the service durability of latex-modified cement materials. These permeability results corroborate previous findings that the addition of finely dispersed fillers or pozzolanic additives densifies the OPC matrix and substantially improves long-term durability under aggressive environments [2,3].

### 2.6. FTIR Analysis

**Figure 9:**
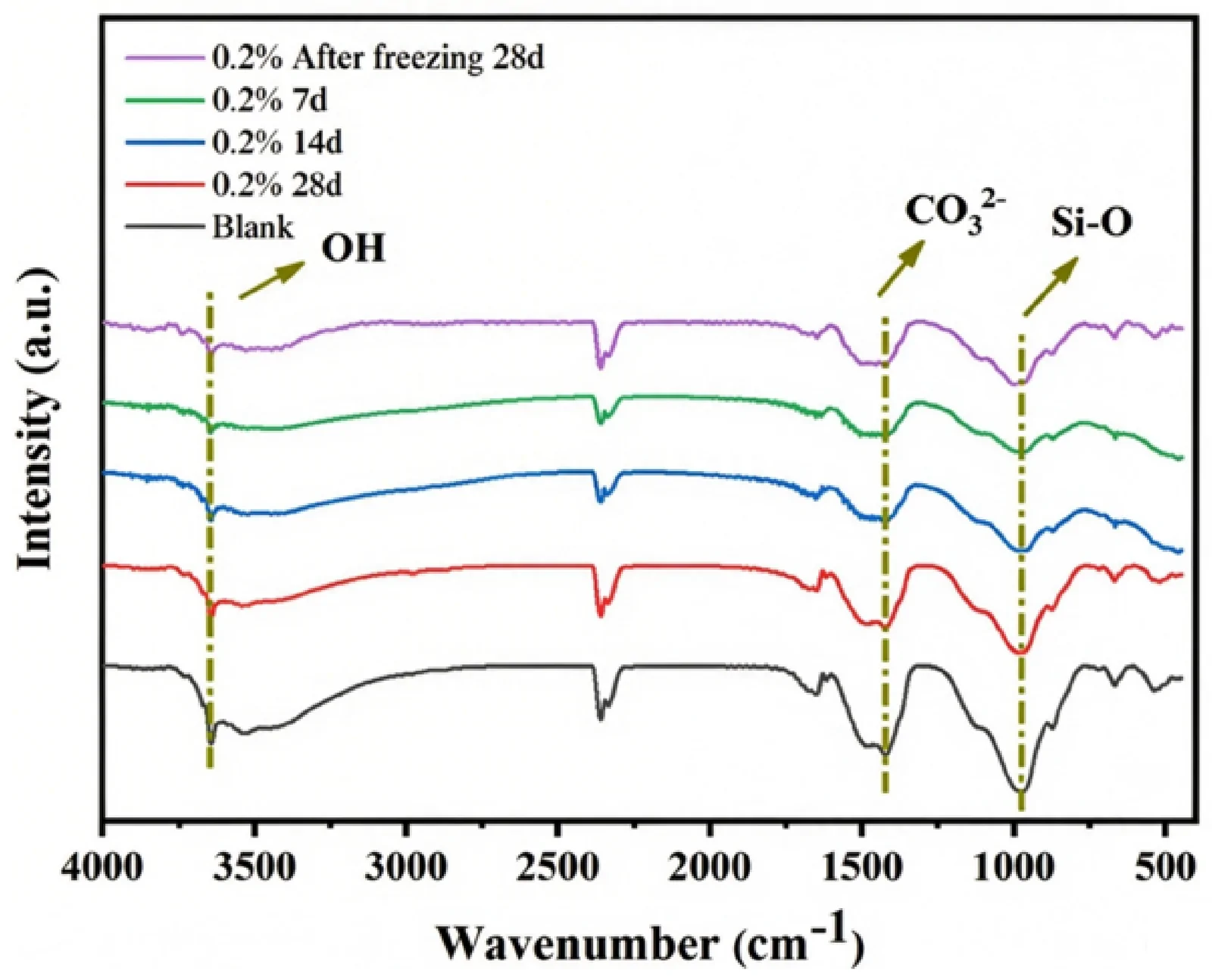
FTIR spectra of cement specimens incorporated with 0.2% latex polymer at different time intervals.

To further reveal the intrinsic chemical modification mechanism of the composite latex polymer on cement-based materials from the molecular perspective, Fourier Transform Infrared Spectroscopy (FTIR) was adopted in this study to analyze the variations in functional groups and hydration product characteristics of latex-modified OPC specimens at different curing ages and before/after freeze–thaw treatment. Figure 9 presents the FTIR spectra of 0.2% latex-modified OPC specimens under different testing conditions.

The results show that the main absorption peak positions of latex-modified OPC are basically consistent with those of plain OPC paste. Typical characteristic peaks appear at 3600 cm^−1^ (–OH stretching vibration), 1500 cm^−1^ (CO_3_^2−^ vibration), and 1000 cm^−1^ (Si–O stretching vibration), and no new characteristic absorption peaks are generated after latex incorporation. This phenomenon indicates that the addition of modified latex does not change the types of cement hydration products or produce new crystalline phases. Nevertheless, the absorption intensity of characteristic peaks gradually increases with the extension of curing time. This variation is mainly because sufficient hydration reactions occur during long-term curing, generating more C-S-H gel and other hydration products, thereby enhancing the absorption intensity of Si–O functional groups. It is further demonstrated that the latex polymer participates in the cement hydration process and interacts with hydration products at the microscopic level, but only regulates the interfacial bonding state of hydration products rather than changing the fundamental chemical composition and phase type of the cement matrix. In addition, the peak positions of functional groups show no obvious deviation before and after 28-day freeze–thaw cycling, which verifies that the latex-modified OPC system maintains excellent chemical structural stability under freeze–thaw erosion.

### 2.7. X-Ray Diffraction Analysis

**Figure 10:**
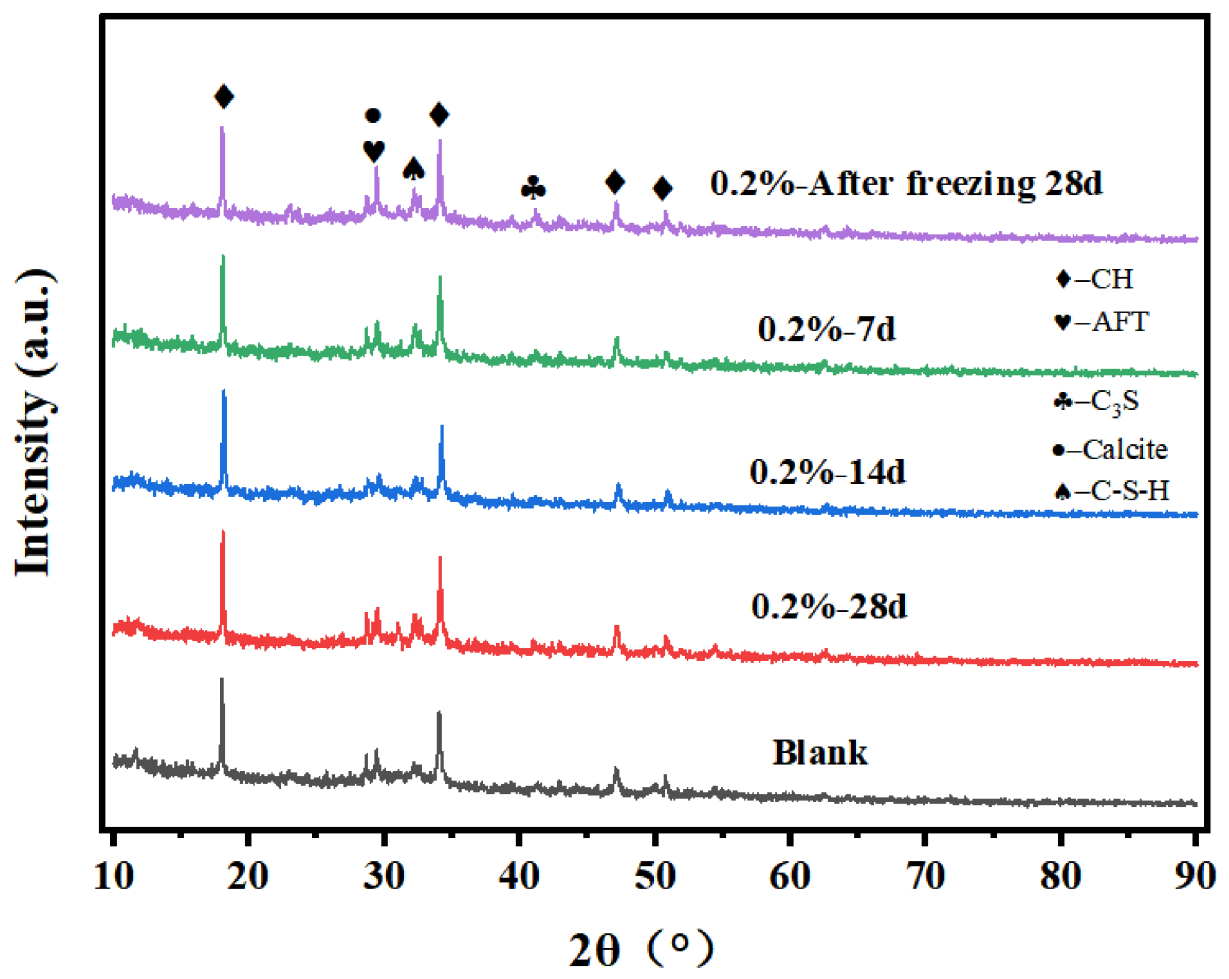
XRD patterns of cement specimens incorporated with 0.2% latex polymer at different curing ages.

To further reveal the microscopic modification mechanism of optimized latex polymer on cement-based materials from the perspective of crystalline phase structure, X-ray Diffraction (XRD) tests were conducted to systematically analyze the variations in phase composition and crystal structure evolution of cement hydration products. The XRD patterns of 0.2% latex-modified OPC specimens at different curing ages are displayed in Figure 10.

It can be observed that the diffraction peak positions of latex-modified OPC specimens are highly consistent with those of plain OPC paste, and no new diffraction peaks appear after latex incorporation. Compared with the control group, the modified OPC specimens exhibit enhanced diffraction intensity of inherent hydration product peaks without any peak shift. These results confirm that the addition of latex polymer does not generate new crystalline phases or change the fundamental phase composition of cement hydration products, and only improves the crystallinity and diffraction response of existing hydration phases.

In particular, the characteristic diffraction peak intensity of calcium silicate hydrate (C-S-H) gel in latex-modified OPC is significantly higher than that in plain OPC paste. This phenomenon verifies that the optimized latex modification can effectively promote the generation and growth of C-S-H hydration products, which is the essential microscopic reason for the improved mechanical performance of modified OPC composites [44]. Moreover, the XRD characteristics of the specimens show no obvious changes before and after 28-day freeze–thaw cycling. It is indicated that long-term freeze–thaw erosion has negligible adverse effects on the crystalline structure and phase composition of latex-modified OPC, further demonstrating the outstanding microscopic phase stability and freeze–thaw resistance of the modified system.

### 2.8. Interaction Mechanism Between Polymer and Cement

**Figure 11:**
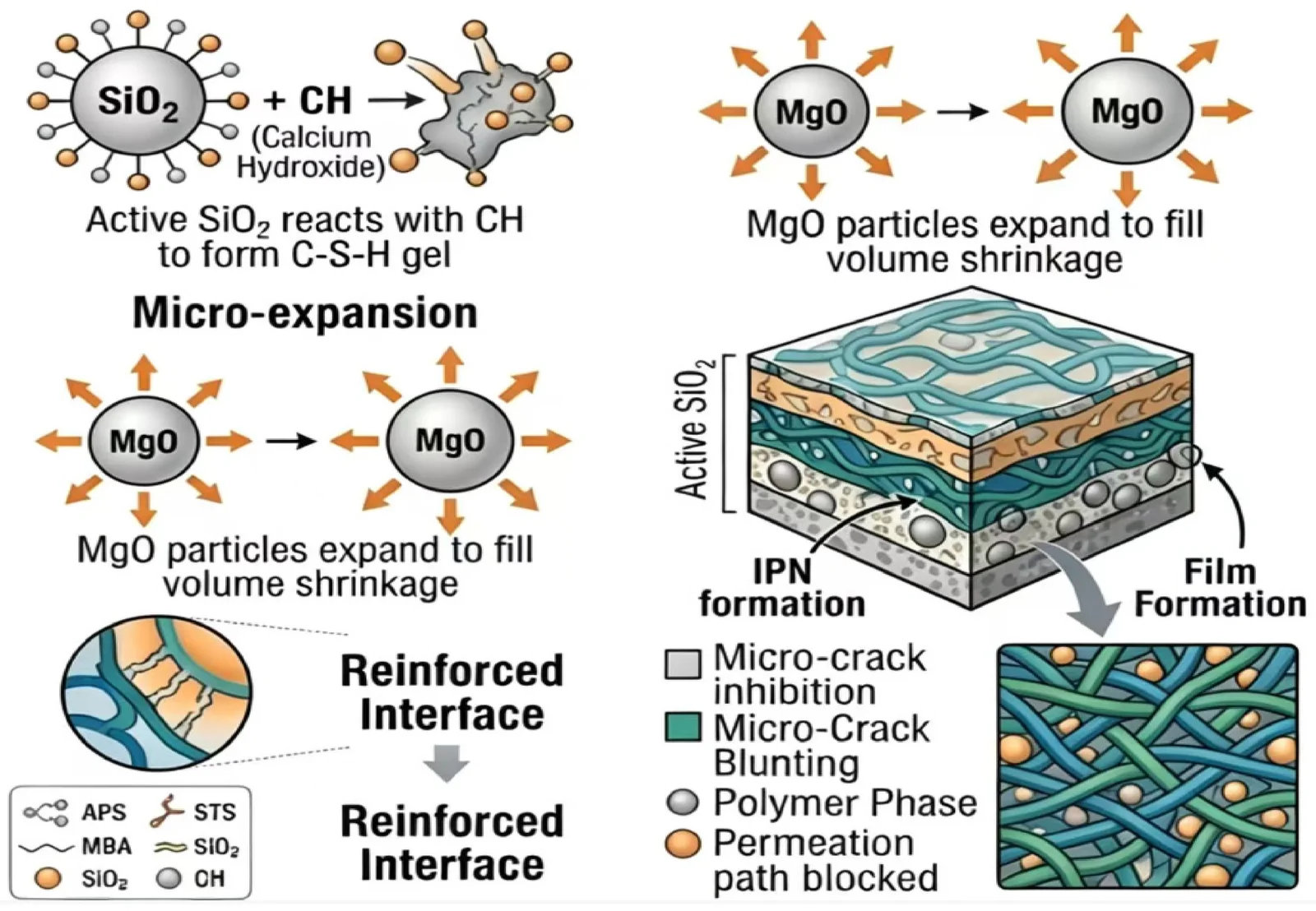
Schematic diagram of the interaction mechanism of the latex polymer in cement.

On the basis of the SEM morphological evidence presented in Section 2.9, the latex polymer-modified cement composites exhibit improved mechanical performance and durability in the face of freeze–thaw cycling, and the microscopic characterizations also confirm the stability of their hydration products and crystal structures. The observed densification of the matrix, the bridging of microcracks by the polymer film, and the preserved compactness after freeze–thaw cycling can be attributed to the unique synergistic interaction between the composite latex and the cement matrix. This synergistic mechanism is summarized schematically in Figure 11, which primarily encompasses multiple aspects including chemical cross-linked network construction, pozzolanic reaction enhancement, interfacial transition zone strengthening, and microcrack inhibition, as discussed in detail below.

At the early stage of OPC hydration, the APS/STS redox initiation system efficiently generates abundant active free radicals at room temperature, which triggers the cross-linking reaction between the MBA cross-linker and natural rubber molecular chains, thereby forming a flexible polymer network structure [45]. Meanwhile, the incorporated reactive nano-SiO_2_ plays a dual modification role: it not only physically reinforces the flexible polymer network but also participates in the OPC hydration reaction. The pozzolanic reaction between reactive SiO_2_ and portlandite from OPC hydration generates additional C-S-H gel [46], which helps refine the microstructure of the interfacial transition zone. It should be noted that, under EN 197-1 [18], the permitted replacement of Portland clinker by silica fume ranges from 6 to 10 wt%, and OPC/silica-fume blend cements must comply with the Frattini test at 8 or 15 days of curing. Silicic pozzolans exhibit unique chemical and physical characteristics, which are critical for their application in cementitious composites [47]. Their pozzolanic reactivity can be evaluated by comparing with commercial silica fume using agro-industrial waste-derived pozzolanic materials [48]. In addition, the addition of silicic pozzolans significantly alters the rheological behaviour of fresh cement pastes [49]. Extensive physicochemical investigations reveal the reaction mechanism of pozzolanic cements exposed to gypsum attack [50]. Long-term studies further confirm the durability performance of silicic pozzolans and blended Portland cements under gypsum-rich aggressive conditions [51]. The reactive nano-SiO_2_ used in this study is introduced at only 5 wt% (as a filler, not as a direct clinker replacement), but because it is chemically much more reactive than silica fume and is purely silicic in both nature and chemical character [16,17], its actual pozzolanic activity is expected to be at least equivalent to, and likely greater than, that of silica fume under the same curing conditions. Furthermore, the synergistic combination of polymer–OPC interaction and pozzolanic reaction is proposed to strengthen the interfacial bonding between the organic polymer phase and inorganic hydration products via physical pore filling and microcrack bridging, which can be visually confirmed from the denser matrix and continuous polymer films observed in SEM images. Additionally, MgO exhibits a moderate micro-expansion effect during hydration, which can effectively compensate for volumetric shrinkage of the OPC matrix at later ages and reduce internal micro-pore defects.

With the continuous progress of OPC hydration and the gradual consumption of free water within the matrix, the cross-linked latex particles dispersed in capillary pores undergo demulsification and progressive film formation. The uniformly formed flexible polymer film interweaves and interpenetrates with rigid OPC hydration products, ultimately constructing a compact organic–inorganic interpenetrating polymer network (IPN) structure [52]. This hierarchical composite structure optimizes the internal pore distribution of the matrix, blocks the permeation paths for water molecules, facilitates effective stress dispersion under external loading, and restrains the initiation, propagation, and blunting of microcracks. However, it must be emphasized that the foregoing mechanistic interpretation of a synergistic interaction among the polymer, OPC, 5 wt% nano-SiO_2_, 3 wt% MgO and MBA (0.031 g per 10 g of latex emulsion) remains a working hypothesis. Although it is physically reasonable on the basis of the available SEM, FTIR and XRD evidence, the existence, type and quantitative degree of any true synergy have not yet been experimentally determined.

To test this hypothesis rigorously, we propose a Frattini test programme performed at 8 and 15 days on the OPC blend cements prepared with and without the following components: (i) 5 wt% nano-SiO_2_ alone, (ii) 3 wt% MgO alone, (iii) MBA alone (0.031 g per 10 g of latex emulsion), (iv) 5 wt% nano-SiO_2_ + 3 wt% MgO, (v) 5 wt% nano-SiO_2_ + MBA (0.031 g per 10 g of latex emulsion), and (vi) 5 wt% nano-SiO_2_ + 3 wt% MgO + MBA (0.031 g per 10 g of latex emulsion). This six-sample matrix will make it possible to determine, specify and quantify the type and degree of synergy actually occurring in the polymer–OPC + 5 wt% nano-SiO_2_ + 3 wt% MgO + MBA system, following the approach previously demonstrated by Talero for the expansive synergy between ettringite formed from pozzolan-derived Al_2_O_3_ and OPC-derived C_3_A in common sulphate media [53,54,55] and for the Friedel’s salt synergy in common chloride media [56]. When the same experimental conditions apply and all other factors are held equal, the pozzolanic activity of the nano-SiO_2_ used here is expected to be at least equal to, and very likely greater than, that of conventional silica fume at the later ages of 8 and/or 15 days [16,17,18]. Finally, because the polymer–OPC interaction may induce shrinkage whereas the hydration of 3 wt% MgO produces expansion, the volume stability of the corresponding paste should also be determined using the EN 196-3:2005 [57] standard method (Le Chatelier needles), preferably after the initial and final setting times have been measured on the same paste. These proposed Frattini and volume-stability programmes, together with the existing microscopic evidence, are intended to provide the quantitative, experimentally grounded validation required to move the present interpretation from a working hypothesis to an established mechanism; they will be implemented in a follow-up study by the same authors. It should also be noted that calcined clays and silica fume/nano-SiO_2_ cannot be simply equated in terms of pozzolanic performance because of their distinct chemical nature (aluminosilicate versus purely silicic character), as discussed in the previous literature [50,51]. Considering the cost-efficiency emphasized in those references, the six-series Frattini-test matrix is treated as our primary experimental validation method, whereas TGA and other micro-scale characterizations are regarded only as secondary complementary supporting measurements.

### 2.9. Microscopic Morphology

**Figure 12:**
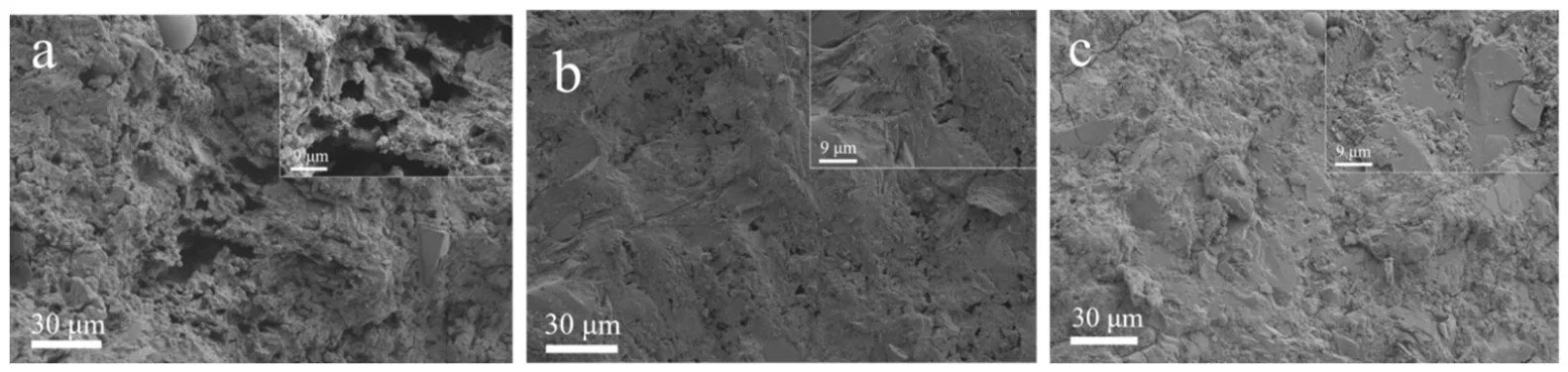
SEM micrographs of cement specimens: (**a**) plain OPC control; (**b**) incorporated with 0.2% latex polymer cured for 14 days; (**c**) incorporated with 0.2% latex polymer cured for 28 days.

**Figure 13:**
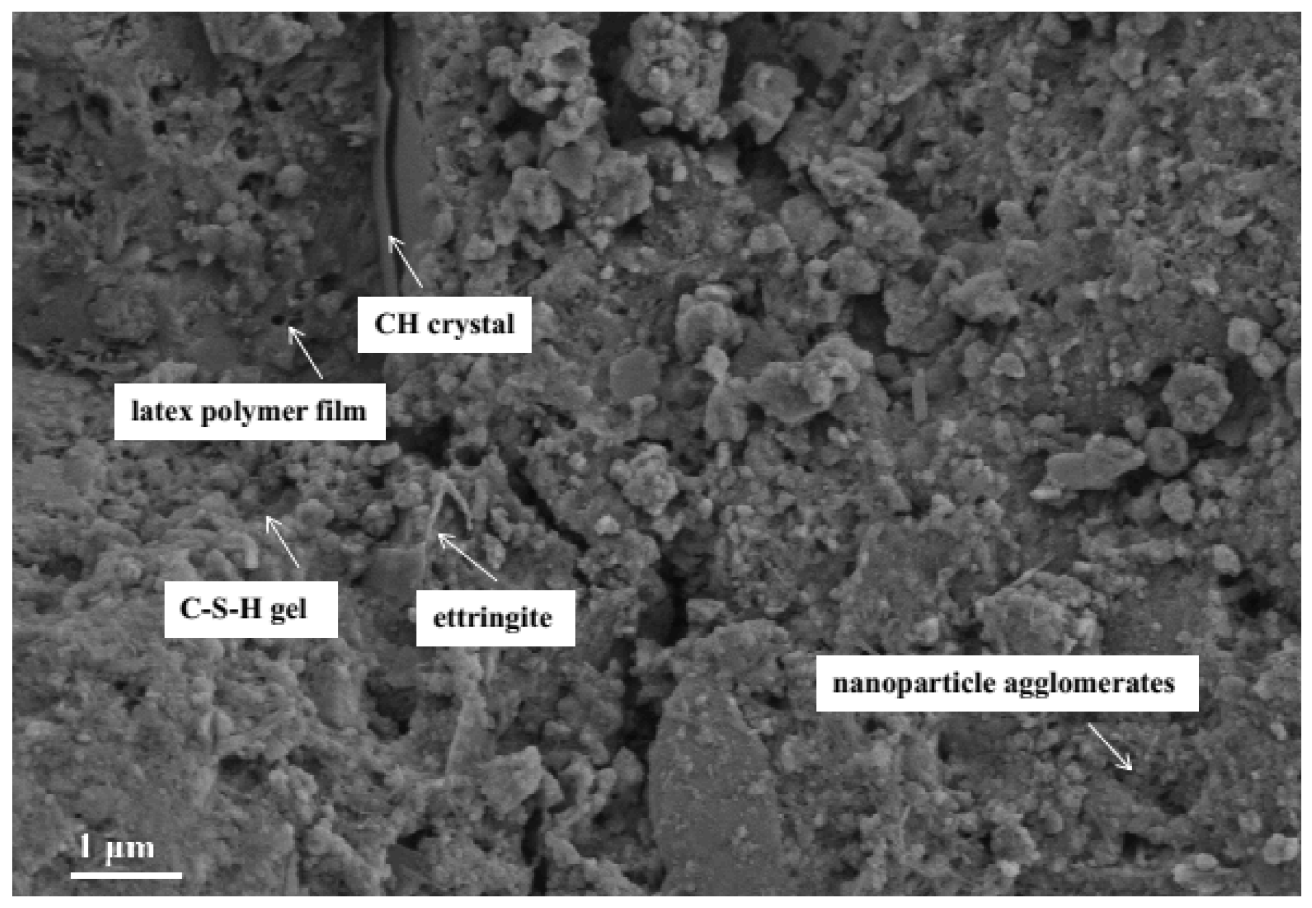
SEM micrograph of the cement paste specimen incorporating 0.2% latex polymer after 28 days of curing.

**Figure 14:**
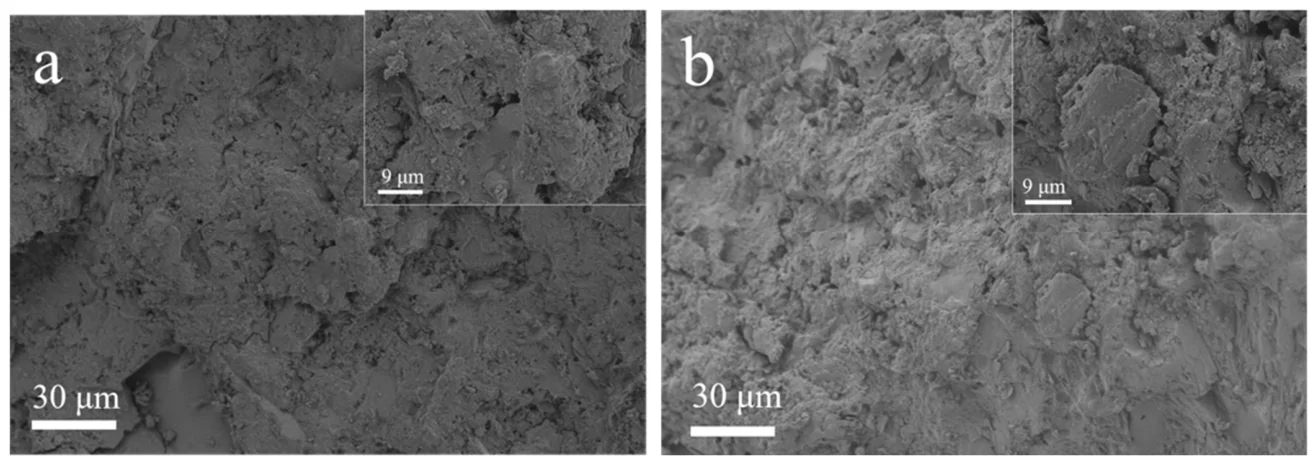
SEM micrographs of the 0.2% latex-modified OPC specimens from the control and freeze–thaw groups: (**a**) after freeze–thaw cycles; (**b**) unfrozen control specimen.

Combined with the foregoing macroscopic durability tests and hydration product characterization, SEM microscopic morphology observation was further performed to visually reveal the structural densification and anti-damage mechanism of latex-modified OPC paste. The microstructural morphologies of plain OPC and 0.2% latex-modified OPC at different curing ages are shown in Figure 12 and Figure 13, while the microscopic structural changes in modified specimens before and after freeze–thaw cycling are displayed in Figure 14.

As shown in Figure 12a, the plain OPC paste presents abundant visible pores and microcracks, and the hydration products are loosely stacked, forming a defective and discontinuous matrix structure. In comparison, the incorporation of 0.2% latex polymer significantly optimizes the microscopic morphology. After 14 days of curing (Figure 12b), the gaps between hydration products are obviously reduced and the matrix compactness is greatly improved. When the curing age extends to 28 days (Figure 12c), the cement matrix achieves the optimal densification state, with no obvious large pores or penetrating cracks observed. This microstructural evolution demonstrates that the appropriate latex dosage can effectively refine pore structure, fill micro-defects, and promote the formation of a dense and continuous cement microstructure, which fundamentally accounts for the improved mechanical strength and impermeability of modified OPC composites.

As shown in Figure 13, the SEM micrograph of cement paste incorporated with 0.2% latex polymer displays a dense and compact composite microstructure. The continuous latex polymer film coats hydration products and lines pore walls, which can effectively bridge microcracks, greatly enhance the interfacial bonding strength between organic phase and inorganic hydration products, and improve the overall structural stability of cement composite materials. In addition, the nano-SiO_2_ agglomerates observed in the matrix act as nucleation sites and participate in pozzolanic reactions to generate extra C-S-H gel, further refining pore structure and densifying the overall microstructure. The arrowed continuous film in Figure 13 was identified as the latex polymer film on the basis of its morphological features (a smooth, flexible layer partially coating hydration products and bridging pore walls) and was further corroborated by EDS/EDX elemental analysis, as described in Section 3.4.7. EDS line scans and area maps showed a pronounced carbon signal along the film contour, together with Si and O signals from the underlying C-S-H/nano-SiO_2_ region and Ca signals from the adjacent OPC hydration products; the spatial distribution of these elements confirms the organic–inorganic composite nature of the film-covered interface.

Figure 14 further compares the microstructural differences in latex-modified OPC before and after freeze–thaw damage. After the freeze–thaw cycles (Figure 14a), the cement matrix generates newly developed interconnected pores and microcracks, leading to a certain degree of structural loosening. However, its overall compactness is still remarkably better than that of the original plain OPC matrix. In contrast, the unfrozen modified specimen (Figure 14b) exhibits densely stacked hydration products with only a small number of isolated micropores, maintaining complete structural integrity. It is indicated that the formed organic–inorganic composite structure can effectively resist the microstructural damage caused by freeze–thaw alternating stress and inhibit crack propagation. This microscopic observation is highly consistent with the foregoing mass loss rate and residual strength test results (Figure 6), further verifying that the optimized latex modification can endow cement-based materials with stable microstructure and improved freeze–thaw resistance within the tested cycle range.

It should be noted that the freeze–thaw tests in the present study were conducted over a limited number of cycles (7 cycles) due to time and sample constraints. As such, the results presented herein reflect only the short-term freeze–thaw performance of the latex-modified OPC-based composites under the specific test conditions employed, and do not permit extrapolation to long-term freeze–thaw durability. For comparison, standard protocols such as ASTM C666 (ASTM International, West Conshohocken, PA, USA) typically require extended cycling (up to 300 cycles), and previous studies [23] have reported performance changes over such extended durations. Accordingly, the conclusions drawn in this work are confined to the observed trends within the limited cycle range. Future work with a larger number of freeze–thaw cycles is necessary to validate the long-term frost resistance of the modified systems.

In addition, XRD, FTIR and SEM were adopted in this study to indirectly characterize the pozzolanic reactivity of reactive nano-SiO_2_ and MBA. Although these microscopic tests can reflect the evolution of hydration products and microstructure, the Frattini test and Le Chatelier paste volume stability assessment serve as more direct and decisive methods for the quantitative evaluation of pozzolanic activity. Therefore, the synergistic pozzolanic effect between reactive nano-SiO_2_ and MBA is inferred from the results of macroscopic mechanical properties and freeze–thaw durability, rather than obtained from direct pozzolanic activity tests. Frattini tests together with Le Chatelier paste volume-stability measurements will be performed in follow-up research to quantitatively characterize the pozzolanic reactivity of this composite system and provide more robust evidence for the synergistic mechanism observed in this work.

## 3. Materials and Methods

### 3.1. Materials

The chemical reagents used in this experiment are as follows: natural latex (analytical grade, Shandong Guangju Biological Technology Co., Ltd., Jinan, China), ammonium persulfate (APS; analytical grade, Sinopharm Chemical Reagent Co., Ltd., Beijing, China), sodium thiosulfate (STS; analytical grade, Sinopharm Chemical Reagent Co., Ltd.), N,N′-methylenebisacrylamide (MBA, analytical grade, Shandong Keyuan Biochemical Co., Ltd., Jinan, China), silicon dioxide (nano-SiO_2_, analytical grade, Xilong Scientific Co., Ltd., Shantou, China; manufacturer-reported median particle size ≈ 20 nm, specific surface area ≈ 200 m^2^/g, purity ≥ 99.5%), and magnesium oxide (reactive MgO, analytical grade, Shandong Keyuan Biochemical Co., Ltd.; manufacturer-reported median particle size ≈ 50 nm, specific surface area ≈ 30 m^2^/g, reactivity index (citric acid neutralization time) ≤ 80 s, purity ≥ 98%).

The cement utilized in this experiment is Ordinary Portland Cement (P·O 42.5), manufactured by Shandong Shanshui Cement Group Co., Ltd., Jinan, China. According to the manufacturer’s technical data sheet for the corresponding clinker, the tricalcium aluminate (C_3_A) content of the clinker exceeds 5 wt%. Accordingly, the binder is classified as Ordinary Portland Cement (OPC, ASTM Type I) in accordance with ASTM C150. (ASTM International, West Conshohocken, PA, USA).The chemical composition of the OPC is detailed in Table 1. Note: The P·O 42.5 cement used in this study is a finished blended cement containing mineral fillers. Therefore, Bogue mineral-phase calculation on these XRF data has not been performed, as this method was originally designed for pure clinker. Quantitative determination of actual mineral phases via XRD-Rietveld refinement was also not performed within the scope of the present experiments; such analysis is recommended for future investigations on separated clinker particles to obtain real-world mineralogical information.

### 3.2. Preparation of Polymer

#### 3.2.1. Preparation of Latex Polymers

**Figure 15:**
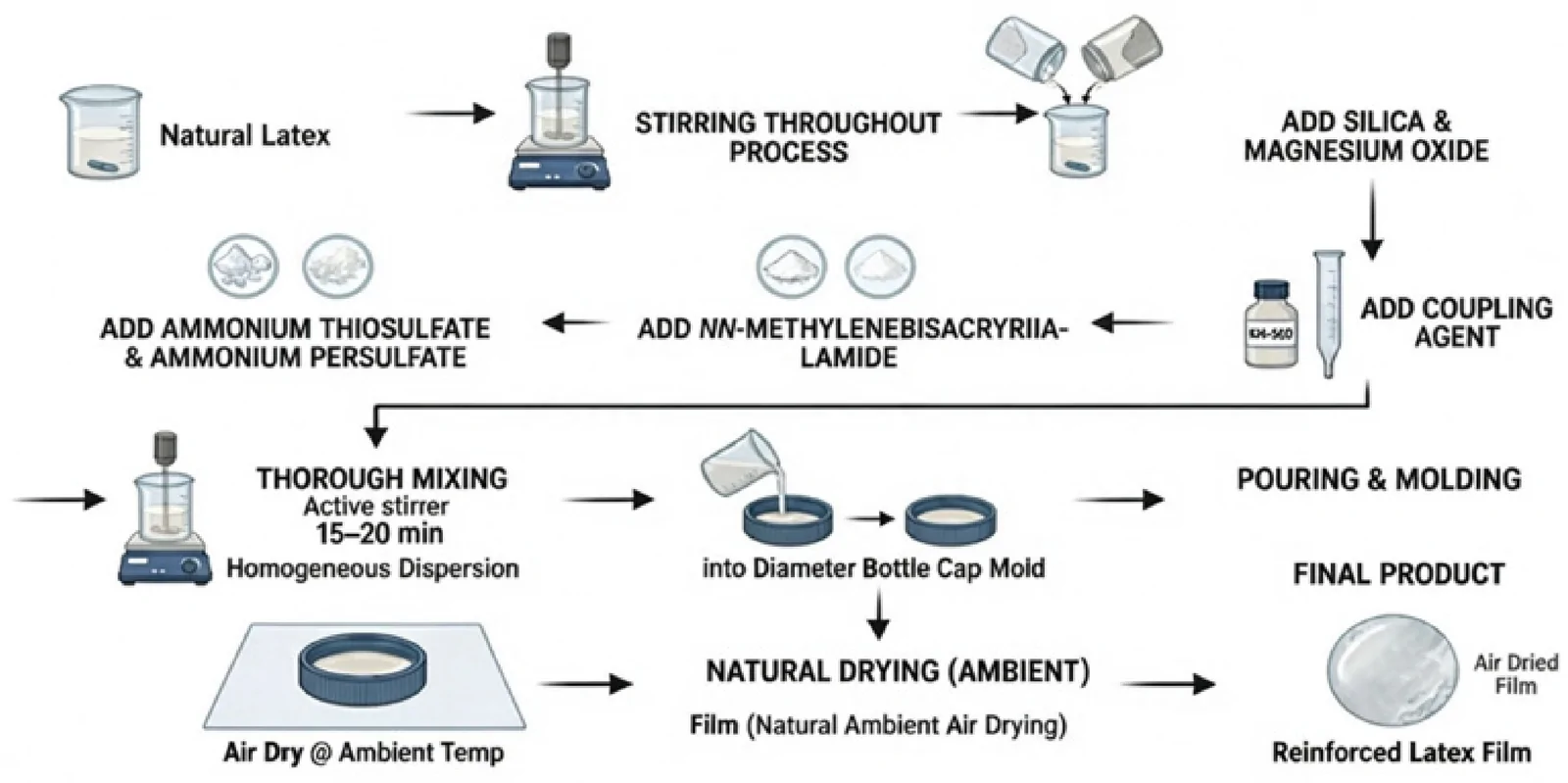
Schematic flowchart of latex polymer preparation.

**Figure 16:**
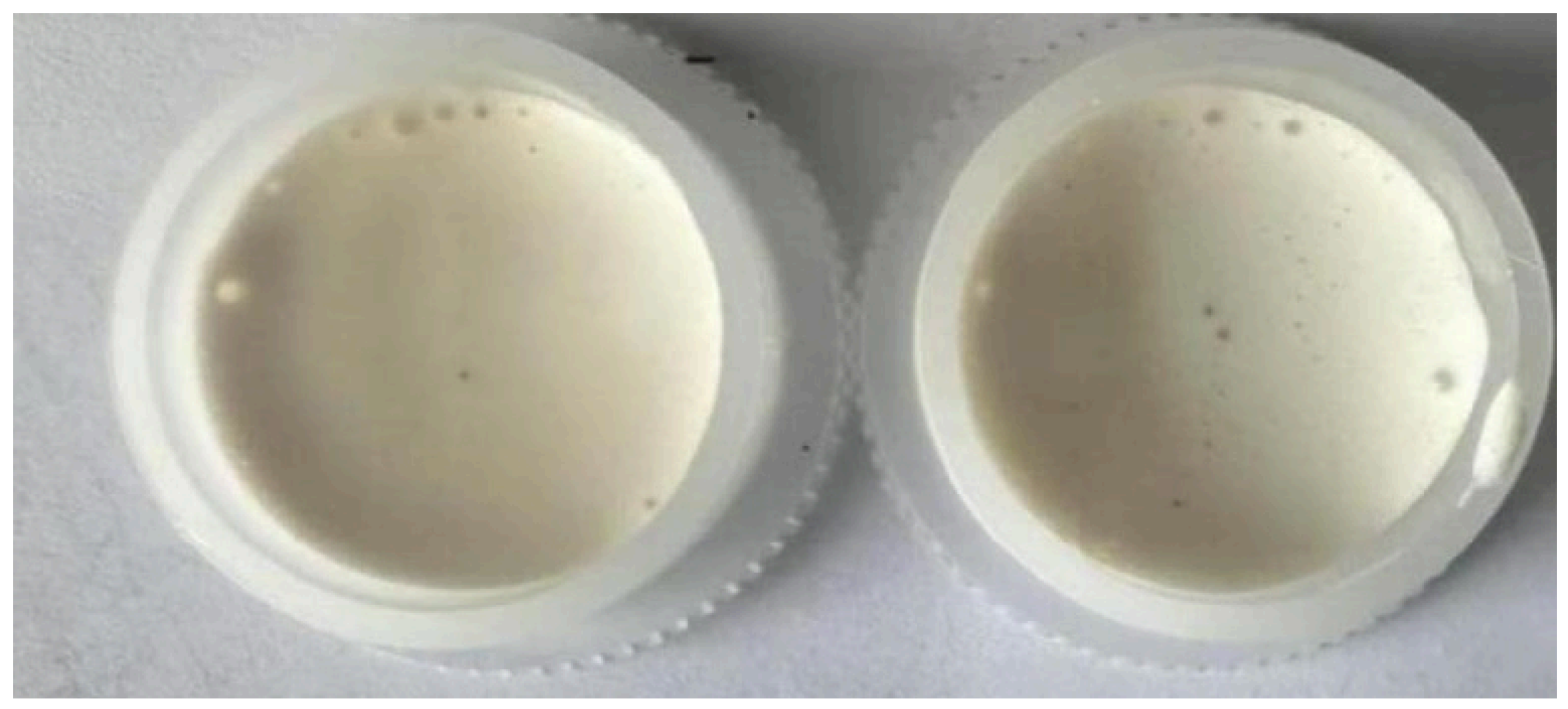
Latex polymer film.

The modified latex polymer was prepared through an ambient-temperature APS/STS redox initiation system, using N,N′-methylenebisacrylamide (MBA) as a cross-linking agent, with reactive nano-SiO_2_ and MgO incorporated as functional reinforcing components. The resulting composite latex was subsequently used as a cement modifier. The preparation involves sequential addition of raw materials, homogenization, and static curing to form a complete polymer film (Figure 15 and Figure 16).

#### 3.2.2. Preparation Procedure

A certain amount of natural rubber latex (NRL) with a solid content of 60% was weighed and placed in a beaker. Under continuous magnetic stirring, magnesium oxide (MgO), silicon dioxide (nano-SiO_2_), N,N′-methylenebisacrylamide (MBA), ammonium persulfate (APS), sodium thiosulfate (STS), etc., were sequentially incorporated into the latex system to prepare the composite polymer emulsion. Specifically, the NRL system was first pre-stabilized for 5 min, after which the optimal dosages of MgO (3 wt% relative to the latex mass), nano-SiO_2_ (5 wt%), and MBA (0.031 g per 10 g of latex emulsion) were added sequentially, with 2 min of homogenization between additions. The APS/STS redox initiator pair was finally introduced to trigger graft copolymerization and cross-linking of the natural rubber molecular chains. Each abbreviation (APS, STS, MBA) is defined in Section 2.1 and listed in Section 3.1 for completeness.

#### 3.2.3. Performance Analysis of the Polymer Film

The freshly prepared composite polymer emulsion was poured into circular molds and cured at room temperature, and the complete polymerization and film-forming time was recorded in real time. A single-factor experimental method was adopted in this study, in which the dosages of SiO_2_ and MgO were adjusted independently while the contents of other components remained unchanged. After full curing, the tensile mechanical properties of polymer films were tested and analyzed.

### 3.3. Preparation of Cement Specimens

#### 3.3.1. Preparation of Cement Paste Specimens

In this test, the water–cement ratio was fixed at 0.4. For each batch, 1350 g of cement and 540 g of deionized water were accurately weighed. The cement paste preparation procedure was conducted as follows: water was first poured into the mixing bowl, and cement powder was subsequently added. After fixing the mixing bowl, the mixer was operated following a standard procedure: low-speed stirring for 2 min, a 15 s interval, and high-speed stirring for another 2 min. After uniform mixing, the fresh cement paste was poured into pre-treated molds. The specimens were vibrated to eliminate internal bubbles and ensure dense filling, and the upper surface was smoothed. All molds were pre-coated with a release agent to facilitate complete demolding without surface adhesion defects.

#### 3.3.2. Preparation of Latex-Modified Cement Specimens

The prepared composite latex polymer emulsion was pre-dispersed in water under stirring to achieve uniform dilution, and then incorporated into fresh cement paste. The mixture was stirred continuously for another 2 min to ensure homogeneous blending, and the obtained modified OPC paste was poured into standard molds for specimen fabrication.

### 3.4. Performance Analysis and Characterization

#### 3.4.1. Mechanical Strength Testing

The mechanical properties of the specimens were evaluated using a microcomputer-controlled automatic flexural and compressive testing machine (Model YAW-300D/10D, Jinan Hengsi Shengda Instrument Co., Ltd., Jinan, China). The specimens, with dimensions of 40 mm × 40 mm × 160 mm, were prepared with a water-to-cement ratio of 0.4 and incorporated with varying dosages of polymer latex. All specimens were cured at 20 ± 2 °C and 95 ± 2% relative humidity for 7 and 28 days prior to testing. The testing procedures strictly followed the GB/T 17671-2021 standard [58]. In accordance with this standard, compressive strength tests were conducted on the two halves of the fractured flexural specimens using a 40 mm × 40 mm loading area. All reported values represent the average of at least three replicates (*n* ≥ 3).

#### 3.4.2. Freeze–Thaw Cycle Testing

Following the standard 28-day curing period, the specimens were prepared for freeze–thaw resistance testing. Two days prior to the test, both the experimental and control specimens were removed from the curing room and fully saturated in water at 15–20 °C, with the water level maintained 20 mm above the specimen surface. After 48 h of water saturation, the specimens were removed. The control group was returned to the curing environment, while the test group was wiped to remove surface moisture, numbered, and weighed. Subsequently, the specimens were frozen at −15 to −20 °C for 8 h and immediately thawed in a water bath at 15–20 °C. Each freeze–thaw cycle lasted no less than 12 h, and the entire process was repeated for 7 cycles over a total duration of 4 days, in accordance with the rapid freeze–thaw method specified in GB/T 50082-2009 [59]. After the final cycle, the specimen surfaces were dried for weight measurement, and the mechanical properties of both the freeze–thaw group and the blank control group were comparatively evaluated.

In addition to the freeze–thaw and water-permeability tests reported here, complementary durability characterizations—including chloride-penetration resistance, sulfate attack resistance, accelerated carbonation, and extended freeze–thaw cycling (up to 300 cycles according to ASTM C666)—are essential to fully evaluate the long-term performance of latex-modified OPC composites. These additional tests are scheduled in our ongoing work, but their results are not yet available for inclusion in the present manuscript.

#### 3.4.3. Setting Time Testing

The cement paste was poured into a circular conical mold (top diameter 70 mm, bottom diameter 80 mm, height 40 mm) and vibrated to remove entrapped air bubbles, followed by leveling the surface with a spatula. The initial and final setting times were determined using a Vicat apparatus (model ZKS-100, Cangzhou Zhonghui Building Materials Instrument Co., Ltd., Cangzhou, China) operating at a temperature of 20 ± 2 °C and relative humidity ≥ 90%, strictly following GB/T 1346-2011 [60]. The initial setting time was determined using the standard Vicat needle. The Vicat needle was positioned to just touch the surface of the cement paste; the screw was then released to allow the needle to sink freely into the paste. The reading was recorded, and the time at which the needle penetration reached approximately 4 mm was defined as the initial setting time [61]. Subsequently, the initial needle was replaced with the final setting needle. The final setting time was defined as the time when the needle failed to penetrate more than 0.5 mm into the paste, leaving only a slight indentation on the surface from the ring attachment.

#### 3.4.4. Water Permeability Analysis

Water permeability was evaluated using a programmable high-precision concrete permeability tester (model HP-4.0, Tianjin Jindalun Building Materials Instrument Co., Ltd., Tianjin, China), following the stepwise pressurization procedure specified in GB/T 50082-2009 [59]. Initially, the concrete specimens, with their lateral surfaces sealed, were pressed into the test molds and securely fastened to the testing stations on the apparatus platform to prevent any water leakage from the bottom. Once configured and activated, the equipment automatically applied water pressure to the bottom of the specimens in a stepwise manner with high precision, according to a pre-set program. During the test, the top surfaces of the specimens were periodically observed for water seepage, allowing for the accurate recording and evaluation of the impermeability grade of the concrete batch. All permeability measurements were performed on three independent replicate specimens (*n* = 3) for each condition, and the reported impermeability pressures represent the mean values. The corresponding error bars in the results section represent the standard deviation of the three replicates.

#### 3.4.5. Fourier-Transform Infrared Spectroscopy (FTIR) Analysis

FTIR analysis was performed using an infrared spectrometer. Specimens with a 0.2% polymer dosage were dried in an oven at 105 °C. A small amount of the sample was ground into a fine powder and prepared via the KBr pellet technique. The absorption spectra were recorded in the wavenumber range of 400–4000 cm^−1^, with a pure KBr pellet serving as the blank control.

#### 3.4.6. X-Ray Diffraction (XRD) Analysis

Fragments from the interior of the 0.2% dosage specimens, collected after mechanical strength testing, were immersed in anhydrous ethanol for 48 h to terminate hydration. The samples were then dried and pulverized into a fine powder. A thin, uniform layer of the powder was applied to a glass slide and analyzed using an X-ray diffractometer. The scanning range was set from 10° to 90° at a scan rate of 10°/min.

#### 3.4.7. Micro-Morphology Analysis

To examine the micro-morphology, specimens of plain cement paste and latex-modified cement were cured for 28 days. Hydration was terminated by immersion in anhydrous ethanol, followed by oven drying. Samples with flat surfaces were selected, and their bottom surfaces were polished to ensure smoothness. The specimens were fixed onto the sample stage using conductive adhesive, with the cross-section facing upwards. After drying in an oven at 78 °C for 24 h, the samples were coated with a thin layer of gold under vacuum and observed using a Field Emission Scanning Electron Microscope (FE-SEM, Merlin Compact, Carl Zeiss AG, Oberkochen, Germany). In addition, representative regions containing the polymer film were analysed by energy-dispersive X-ray spectroscopy (EDS/EDX) to obtain elemental maps and line scans for C, O, Si, Ca and Mg, in order to distinguish the organic polymer film from inorganic hydration products and to confirm the distribution of the nano-SiO_2_ and MgO fillers.

## 4. Conclusions

In this study, a composite latex polymer for cement modification was successfully prepared. The incorporation of the latex polymer achieves favorable synergistic modification effects on the mechanical performance, microstructure, and short-term freeze–thaw resistance of OPC paste. Macroscopic test results verify that the optimal 0.2% latex dosage effectively enhances the mechanical strength of OPC-based materials. Compared with plain OPC paste, the flexural strength and compressive strength of latex-modified cement are increased by 16% and 18%, respectively.

Chemical and microscopic characterizations systematically reveal the intrinsic modification mechanism of latex polymer. FTIR analysis confirms that latex modification does not alter the basic chemical functional group structure of OPC hydration products, proving excellent chemical compatibility between organic polymer and inorganic OPC matrix. XRD test results further indicate that latex incorporation significantly improves the diffraction intensity of C-S-H gel, demonstrating that the polymer can effectively promote the generation and development of OPC hydration products and optimize the hydration reaction degree. SEM morphology observations intuitively show that the latex polymer can fill internal micro-pores and microcracks of the OPC matrix, refine pore structure defects, and construct a dense and continuous organic–inorganic composite microstructure. The optimized compact microstructure reduces internal stress concentration and structural defects, which fundamentally accounts for the improved mechanical performance of modified OPC-based materials.

Moreover, the organic–inorganic composite network structure formed by latex modification endows the OPC matrix with good structural stability. After the freeze–thaw cycles employed in this study, the modified specimens maintain complete microscopic morphology and low mass loss, indicating that the latex polymer can effectively resist freeze–thaw-induced microstructural damage and improve the frost resistance within the tested conditions.

In conclusion, the self-prepared composite latex polymer exhibits reliable modification performance for OPC-based materials within the limited experimental scope of this study. It improves the mechanical properties and short-term freeze–thaw resistance of OPC paste at the optimal dosage of 0.2 wt%. However, any statement that the system synergistically optimizes OPC hydration behavior and freeze–thaw resistance should be regarded as a working hypothesis rather than an established conclusion, because the type and degree of synergy among the polymer, OPC, nano-SiO_2_, MgO and MBA have not yet been quantified. Further validation by the Frattini test programme and the EN 196-3:2005 volume-stability measurement proposed in Section 2.9 is therefore necessary. This study provides a feasible experimental basis and technical reference for the research and engineering application of high-performance OPC admixtures.

## Figures and Tables

**Table 1:** Chemical Composition of Cement.

Cement Composition	Loss	SiO_2_	Al_2_O_3_	Fe_2_O_3_	CaO	MgO	SO_3_
P·O 42.5	5.61	22.75	8.66	2.77	51.54	4.91	1.87

Footnote: Minor oxides including K_2_O, Na_2_O, TiO_2_ and MnO were also present in cement as trace components, which were not detected and listed in this table.

## Data Availability

The data presented in this study are available in the article.
